# Free circulating ICAM-1 in serum and cerebrospinal fluid of HIV-1 infected patients correlate with TNF-α and blood-brain barrier damage

**DOI:** 10.1155/S0962935192000486

**Published:** 1992

**Authors:** M. K. Sharief, M. Ciardi, M. A. Noori, E. J. Thompson, A. Salotti, F. Sorice, F. Rossi, A. Cirelli

**Affiliations:** 1 Department of Neurochemistry Institute of Neurology The National Hospital for Neurology and Neurosurgery Queen Square London WC1N 3BG UK; 2 The Royal London Hospital Whitechapel London UK; 3 Institute of Infectious Diseases University of Rome “La Sapienza” Rome 00161 Italy; 4 Department of Infectious Diseases University of Pisa Pisa 2013 Italy

## Abstract

The mechanism for the initiation of blood-brain barrier damage and intrathecal inflammation in patients infected with the human immunodeficiency virus (HIV) is poorly understood. We have recently reported that tumour necrosis factor-α (TNF-α) mediates active neural inflammation and blood-brain barrier damage in HIV-1 infection. Stimulation of endothelial cells by TNF-α induces the expression of intercellular adhesion molecule-1 (ICAM-1), which is an important early marker of immune activation and response. We report herein for the first time the detection of high levels of free circulating ICAM-1 in serum and cerebrospinal fluid of patients with HIV-1 infection. Free circulating ICAM-1 in these patients correlated with TNF-α concentrations and with the degree of blood-brain barrier damage and were detected predominantly in patients with neurologic involvement. These findings have important implications for the understanding and investigation of the intrathecal inflammatory response in HIV-1 infection.


Mediators of Inflammation, 1, 323–328 (1992)

### Free circulating ICAM-1 in serum and cerebrospinal fluid of HIV-1 infected patients correlate with TNF-$\alpha$ and blood-brain barrier damage

M. K. Sharief,[1,CA] M. Ciardi,[1] M. A. Noori,[2] E. J. Thompson,[1] A. Salotti,[3] F. Sorice,[3] F. Rossi[3] and A. Cirelli[4]

[1] Department of Neurochemistry, Institute of Neurology, The National Hospital for Neurology and Neurosurgery, Queen Square, London WC1N 3BG, UK;
[2] The Royal London Hospital, Whitechapel, London, UK;
[3] Institute of Infectious Diseases, University of Rome "La Sapienza", 00161 Rome, Italy;
[4] Department of Infectious Diseases, University of Pisa, 2013 Pisa, Italy

[CA] Corresponding Author

**THE mechanism for the initiation of blood-brain barrier damage and intrathecal inflammation in patients infected with the human immunodeficiency virus (HIV) is poorly understood. We have recently reported that tumour necrosis factor-$\alpha$ (TNF-$\alpha$) mediates active neural inflammation and blood-brain barrier damage in HIV-1 infection. Stimulation of endothelial cells by TNF-$\alpha$ induces the expression of intercellular adhesion molecule-1 (ICAM-1), which is an important early marker of immune activation and response. We report herein for the first time the detection of high levels of free circulating ICAM-1 in serum and cerebrospinal fluid of patients with HIV-1 infection. Free circulating ICAM-1 in these patients correlated with TNF-$\alpha$ concentrations and with the degree of blood-brain barrier damage and were detected predominantly in patients with neurologic involvement. These findings have important implications for the understanding and investigation of the intrathecal inflammatory response in HIV-1 infection.**

**Key words:** Blood-brain barrier, Brain inflammation, HIV-1 infection, Intercellular adhesion molecule-1, Tumour necrosis factor

### Introduction

Neurologic involvement is a frequent feature of human immunodeficiency virus (HIV) infection[1] and may be the initial presentation in about 10% of patients.[2] There is increasing evidence that indirect mechanisms, such as the release of cytokines, play an important role in mediating brain inflammation in HIV infection. Indeed it is now widely acknowledged that tumour necrosis factor-$\alpha$ (TNF-$\alpha$), a central mediator of inflammation, plays a crucial role in the development of acquired immunodeficiency syndrome (AIDS).[3] TNF-$\alpha$ is implicated in the pathogenesis of most clinical and pathologic features of AIDS[3] and selectively kills HIV-infected cells, probably through a direct cytotoxic effect. Furthermore, TNF-$\alpha$ enhances the replication of HIV and induces the expression of a wide array of inflammatory cytokines.

Damage to cerebral endothelial cells and blood-brain barriers is another important pathologic feature that contributes to brain damage in HIV infection. Neuropathologic studies have clearly demonstrated that cerebral vasculitis is an early and frequent pathologic feature in HIV seropositive subjects.[4] Such vascular inflammation seems to be immune mediated[4,5] and could be connected to the release of inflammatory mediators. Evidence has been presented that TNF-$\alpha$ induces inflammatory changes on human cerebral endothelial cells.[6,7] Moreover, we have recently shown in a different series of patients that TNF-$\alpha$ mediates blood-brain barrier damage in HIV infected patients.[8] However, the precise mechanism of the TNF-$\alpha$ mediated endothelial damage and the mechanism by which circulating immune cells recognize the HIV infected cells, including cerebral endothelium, are not well understood.

Adhesion of inflammatory cells to vascular endothelium is essential for their migration into inflamed tissues. Endothelial cells lining the postcapillary venules and microcirculation elaborate several adhesion molecules both constitutively and in response to a wide range of inflammatory mediators.[9,10] Detection of adhesion molecules expression *in vivo* at sites of acute immunologic inflammation[11] has been used to infer that functional activation of the endothelium may be occurring at these sites.

Intracellular adhesion molecule-1 (ICAM-1,

© 1992 Rapid Communications of Oxford Ltd

CD54), a molecule bound to the cell surface membrane, is an important early marker of immune activation and response[12,13] including the production of inflammatory vascular injury *in vivo*.[14] Although the expression of ICAM-1, which may confer adhesivity for lymphocytes is most common in haematopoietic tissues, it is also detected in several organs, including the central nervous system (CNS).[15] The presence of free circulating ICAM-1 (cICAM-1) has been recently documented in human sera[16,17] and the expression of ICAM-1 is upregulated by TNF-α and other cytokines.[18] There is also increasing evidence that adhesion receptors can play a significant role in the pathogenesis of AIDS (reviewed by Koopman and Pals[19]).

In this study, serum and CSF samples from HIV infected patients have been examined for the presence of free cICAM-1, as an *in vivo* marker of endothelial cell activation. It is believed this is the first time that circulating ICAM-1 has been shown to be present in serum and CSF from HIV infected patients and that it correlates with both TNF-α levels and the degree of blood-brain barrier damage.

### Patients and Methods

*Patients:* Paired CSF and serum samples were obtained from 37 HIV type-1 (HIV-1) seropositive patients (28 males, 9 females; median age 27.6 years, age range 19–49 years). Patients were classified according to the guidelines of the Centres for Disease control[20] and their clinical features are presented in Table 1.

Cerebrospinal fluid was obtained by a lumbar puncture and cells were separated by cytocentrifugation then all samples were filtered through a 0.45 μm disposable sterile filter (Millipore, Harrow, UK) to remove contaminating particulate materials. The CSF was concentrated by means of Minicon CS15 concentrators (Amicon, Upper Mill, UK). Samples were frozen in aliquots at −70°C and thawed just before use; repeated thawing and refreezing was avoided. Neurosyphilis, frequently associated with HIV infection, was excluded in all patients by fluorescent treponemal antibody absorption and treponemal haemoagglutination tests.

**Table 1**. CDC classification of 37 HIV-1 seropositive patients included in the study

| | No. of patients | Clinical features |
|---|---|---|
| Group II | 2 | Asymptomatic HIV-1 infection |
| Group III | 4 | Persistent generalized lymphadenopathy |
| Group IV | | |
| Subgroup A | 5 | Constitutional disease |
| Subgroup B | 8 | Neurological disease |
| Subgroup C | | |
| Category C-1 | 6 | Specified secondary infections within the CNS |
| Category C-2 | 9 | Other specified secondary infections |
| Subgroup D | 3 | Secondary cancers |

*Controls:* Control CSF and serum samples were obtained from 30 HIV-1 seronegative patients with various non-inflammatory neurologic diseases in whom signs of blood-brain barrier damage were detected at presentation. They included eight patients with meningioma, four with craniopharyngioma, five with intracranial arteriovenous malformation, six with cerebrovascular diseases, three with benign intracranial hypertension, and four with obstructive hydrocephalus. Paired samples were also obtained from 18 normal subjects (median age 32.5 years, range 16–54 years) to determine reference ranges. These subjects presented with nonspecific complaints such as headache or blurring of vision and neurologic examination as well as detailed investigations had excluded a specific cause of their symptoms.

*Detection of cICAM-1:* Measurement of circulating immunoreactive ICAM-1 in serum and CSF samples was performed by a sensitive dot blot analysis[16] with minor modifications. In brief, 1:50 diluted serum and CSF samples concentrated to the same albumin level were spotted onto polyvinyl difluoride membrane (Immobilon, Millipore, Harrow). After nonspecific blocking and washing, the blots were incubated with a monoclonal antibody to ICAM-1[21] and subsequently incubated with a peroxidase-conjugated $F(ab')_2$ fragment (Sigma, St Louis, MO, USA). The blots were then developed with a chromogen solution containing *o*-phenylenediamine dihydrochloride and 100 μl $H_2O_2$ in 100 ml of 0.02 M acetate buffer. As controls, blots were incubated without the first antibody and with a Mab (anti-CD18) against the common beta chain of LFA-1 and Mac-1.

Quantitative analysis of the blots was performed by densitometric evaluation as recently described[22] using a Joyce Loebl Chromoscan 3 densitometric scanner. The intensity of the colour reaction of blots is dependent on the ICAM-1 concentration in the test samples. The colour intensity was measured as reflected light and the maximum absorption was determined at 492 nm. The lower limit of the detection of cICAM-1 is 8 ng/ml.

*Other assays:* Levels of TNF-α in the test samples were determined by a sandwich-type enzyme-linked immunoassay (ELISA) described previously.[23] A standard curve was run on each ELISA plate using recombinant human TNF-α in serial dilutions. Albumin concentrations in CSF and serum samples were measured by electroimmunoassay. The in-

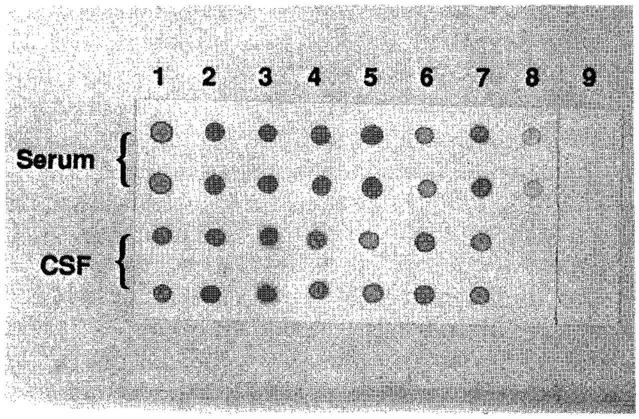

FIG. 1. Dot blots showing the presence of cICAM-1 in serum and CSF; each sample was analysed in duplicate. Lanes 1 to 7 = samples from patients with HIV-1 infection; lane 8 = samples from a healthy subject; and lane 9 = blots incubated with monoclonal antibodies against CD18 to serve as control.

tegrity of the blood-brain barrier was assessed by calculating CSF to serum albumin quotient ($Q_{alb}$)[24] and the degree of barrier damage was graduated according to $Q_{alb}$ as already described.[25]

### Results

*Distribution of cICAM-1:* A variable range of intensities of free cICAM-1 was detected in serum from the control healthy individuals (Figure 1); however, no cICAM-1 reactivity was seen in their CSF. The mean $\pm 2$ SD of maximum absorbance of cICAM-1 in serum from healthy controls (19.7 + 14.6%) was calculated as the cut-off value for determining abnormally high amounts of cICAM-1 in the study population.

High cICAM-1 levels (above the cut-off value in normal controls) were detected in serum of 25 HIV-1 seropositive patients and 13 seronegative controls, whereas cICAM-1 in CSF was seen predominantly in HIV-1 serpositive pâtients (Table 2). Serum cICAM-1 in HIV-1 seropositive patients correlated with corresponding CSF amounts ($r = 0.61$, $p < 0.001$). Free cICAM-1 was detected in CSF of all HIV-1 seropositive patients with neurologic involvement but was not detected in CDC groups II or III; however, cICAM-1 was also detected in CSF of group IV-A patients who showed no clinical evidence of neurologic disease.

*Correlation of cICAM-1 with TNF-α levels:* As shown in Table 2, high TNF-α levels were seen in serum of 20 and in CSF of 22 HIV-1 seropositive patients. There was a strong association between TNF-α and cICAM-1 in the test samples. Of the 22 seropositive patients who had high CSF TNF-α levels, 17 (77%) had high CSF cICAM-1 levels, whereas only two (13%) of 15 patients with no detectable TNF-α in CSF had high CSF cICAM-1 levels. Similarly, 18 (90%) of the 20 patients with high serum levels of TNF-α had high levels of serum cICAM-1, and only two (10%) patients with high serum TNF-α levels had normal serum cICAM-1 levels.

The 20 seropositive patients with high serum TNF-α had higher levels of cICAM-1 in serum (mean absorbance $\pm$ SD = 69.5 $\pm$ 20.9%) compared to those who had no detectable serum TNF-α (mean absorbance = 36.1 $\pm$ 18.8%, $p < 0.01$). Similarly, CSF levels of cICAM-1 (mean absorbance = 54.2 $\pm$ 30.2%) in HIV-1 seropositive patients who had high CSF TNF-α were significantly higher than CSF cICAM-1 in patients with no detectable TNF-α in CSF (mean absorbance = 22.9 $\pm$ 20.8%, $p < 0.001$). Moreover, individual levels of TNF-α in HIV-1 seropositive patients significantly correlated with cICAM-1 in both serum and CSF (Figure 2).

No correlation between TNF-α and cICAM-1 was observed in the HIV-1 seronegative controls. High TNF-α levels were detected in CSF of three seronegative controls who had no cICAM-1 in CSF and six of the 13 seronegative controls with high serum cICAM-1 levels had no measurable serum TNF-α levels. Furthermore, mean absorbance of serum cICAM-1 (48.4 $\pm$ 12.7%) and CSF (17.3 $\pm$ 11.4%) in seronegative controls with detectable TNF-α levels were not significantly different from those with no measurable TNF-α (mean absorbance = 31.2 $\pm$ 14.1% and 13.2 $\pm$ 12.6% respectively).

*Correlation of cICAM-1 with blood-brain barrier damage:* Twenty-four HIV-1 seropositive patients had high $Q_{alb}$ values suggestive of blood-brain barrier damage. All HIV-1 seropositive patients who had

**Table 2.** Distribution of cICAM-1 and TNF-α levels in 37 HIV-1 seropositive patients and 30 seronegative controls showing those with abnormally high values

| Variable | HIV-1 seropositive patients | | HIV-1 seronegative controls | |
|---|---|---|---|---|
| | Median (abnormal) | Range | Median (abnormal) | Range |
| Serum cICAM-1 (% absorbance) | 53.2 (25) | 6–97 | 38.1 (13) | 4–73 |
| CSF cICAM-1 (% absorbance) | 42.7 (19) | 0–98 | 9.4 (5) | 0–53 |
| Serum TNF-α (unit/ml) | 54.8 (20) | 4.5–175 | 40.2 (8) | 2–123 |
| CSF TNF-α (unit/ml) | 47.3 (22) | 0–163 | 31.8 (5) | 0–87 |

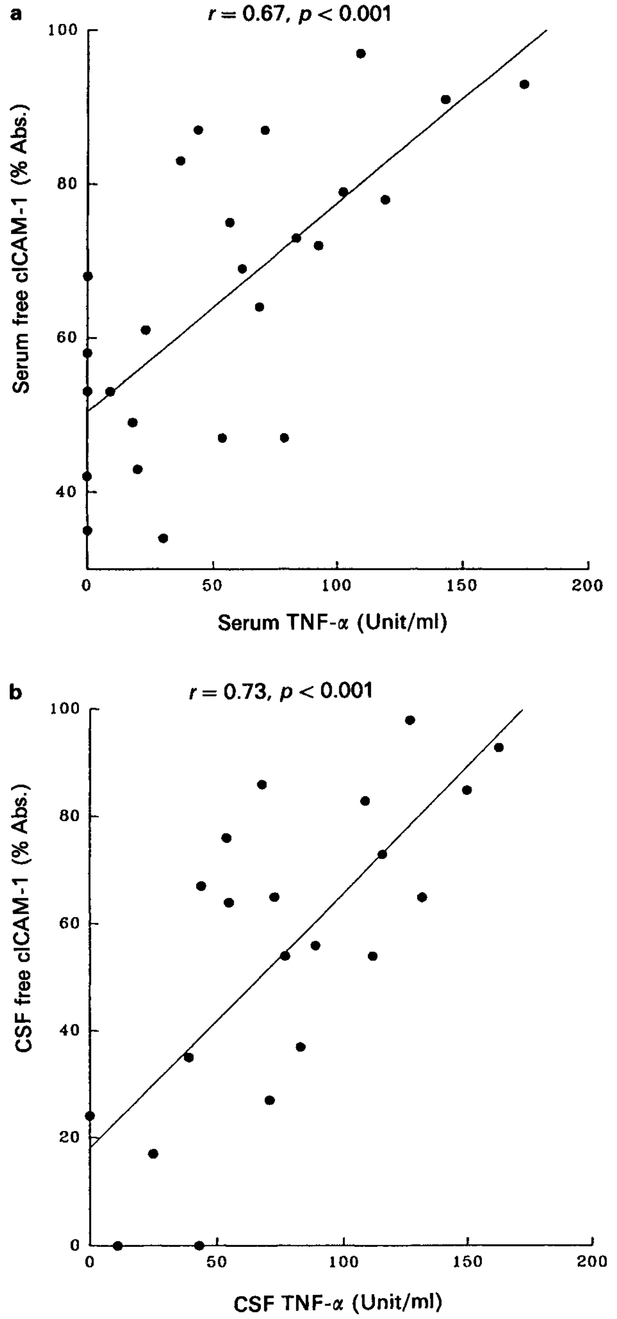

FIG. 2. (a) Correlation of cICAM-1 with TNF-α levels in the serum of HIV-1 seropositive patients. Patients with both normal levels of cICAM-1 and absent TNF-α are not shown. (b) Correlation of cICAM-1 with TNF-α levels in the CSF of HIV-1 seropositive patients. Patients with no measurable cICAM-1 and TNF-α in CSF are not shown.

abnormally high CSF cICAM-1 demonstrated high $Q_{alb}$ values (mean $Q_{alb} = 10.8 \pm 3.7$) indicative of blood-brain barrier damage (Figure 3), whereas only five patients (28%) with no detectable cICAM-1 in CSF showed evidence of barrier damage (mean $Q_{alb} = 6.1 \pm 2.8$, $p < 0.01$). Similarly, high levels of cICAM-1 in serum of HIV-1 seropositive patients were associated with significantly higher incidence of blood-brain barrier

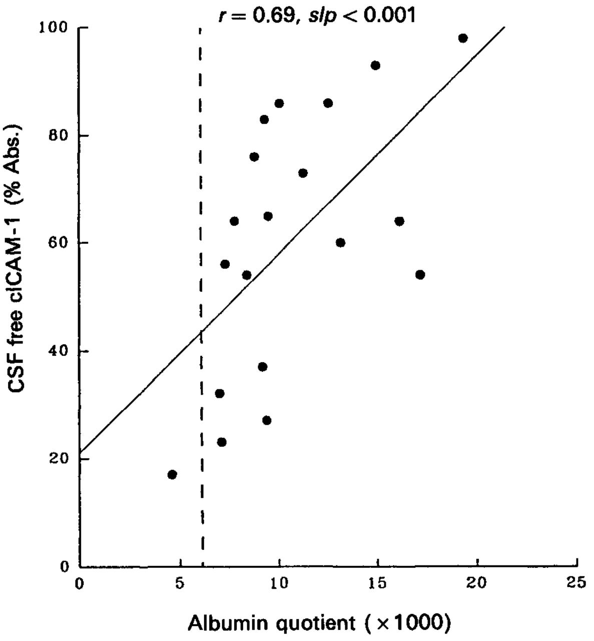

FIG. 3. Correlation of CSF cICAM-1 with CSF to serum albumin quotient in HIV-1 seropositive patients. Those with undetectable cICAM-1 in CSF are not included.

damage (corrected $\chi^2 = 6.8$, $p < 0.05$). Moreover, levels of cICAM-1 in serum and CSF of HIV-1 seropositive patients correlated with the degree of barrier damage (Figure 4). In contrast, the degree of blood-brain barrier damage in seronegative controls did not correlate with either serum or CSF levels of cICAM-1.

### Discussion

In this study of HIV-1 seropositive patients who have relatively high levels of cICAM-1 in serum and CSF, a correlation with both serum and CSF TNF-α levels and the degree of blood-brain barrier damage was observed. It is thought that this represents the

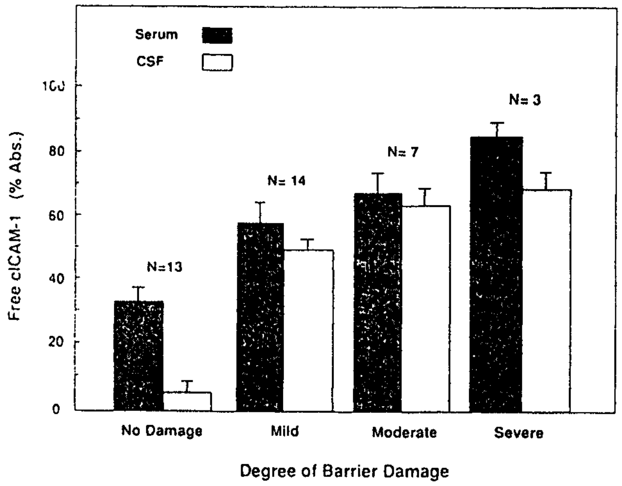

FIG. 4. Correlation of CSF and serum cICAM-1 reactivity with the degree of blood-brain barrier damage in 37 HIV-1 seropositive patients.

first demonstration of a free circulating endothelial cell–leucocyte adhesion molecule in HIV-1 infection. The detection of cICAM-1 raises the question of whether circulating levels of this molecule reflect the amount of ICAM-1 that is bound to the cell surface membrane and pathologic studies are clearly required to evaluate the importance of cICAM-1 in HIV-1 infection.

The presence of high levels of free circulating ICAM-1 in patients with HIV-1 infections also raises the question of whether this molecule carries out biological functions similar to its cell surface bound counterpart, such as the regulation of the intercellular adhesion process. Recent evidence[17] suggests that cICAM-1 retains almost all extracellular domains of membrane ICAM-1 and most structural features necessary for binding to the adhesion receptor lymphocyte function associated antigen 1. Thus, further characterization of cICAM-1 may provide insight into the pathophysiology of inflammatory CNS involvement during HIV-1 infection, particularly the impairment of the blood-brain barrier.

The blood-brain barrier, which is formed by specialized endothelial cells, regulates the interaction between the immune and the central nervous systems. In the normal brain there is very limited lymphocyte traffic, but lymphocyte infiltration is critical for the pathogenesis of several brain diseases.[26,27] A crucial early step in mounting an effective inflammatory or immune response is the promotion of leucocyte adhesion to the vascular endothelium before they can migrate chemotactically to the appropriate microenvironment.[9] ICAM-1 is highly inducible on various cell types, particularly endothelial cells, during inflammation and in response to proinflammatory cytokines, which suggests that ICAM-1 upregulation and cell surface expression are important in the regulation of immune responses.[28]

It has been reported recently that the expression of ICAM-1 is important in leucocyte homing and adhesion to the blood-brain barrier during active stages of experimental autoimmune demyelination.[29] Similarly, the expression of adhesion molecules at the blood-brain barrier has been demonstrated recently in humans.[30,31] Since neurologic involvement in HIV-1 infection is commonly associated with cerebral endothelial and blood-brain barrier damage,[32–34] which can be mediated by TNF-α,[8] it is reasonable to suggest that ICAM-1 may act as a homing signal in HIV-1 related CNS inflammation. In addition, the adhesive interactions between inflammatory cells and the functional adhesion molecules expressed on endothelial cells may result in the activation of inflammatory cells prior to their migration into the intrathecal compartment. The lack of correlation between cICAM-1 and non-inflammatory blood-brain barrier damage observed in the seronegative controls further underlies the potential importance of ICAM-1 in the regulation of CNS inflammation. Whether the release of cICAM-1 preceded or occurred during damage to the blood-brain barrier is not yet clear but will be the subject of further studies.

Free cICAM-1 was detected in CSF of all HIV-1 seropositive patients with neurologic involvement but was absent from CSF of patients with early HIV-1 infection (i.e. CDC groups II and III) who had no evidence of neurologic disease. In this regard, cICAM-1 could be a useful marker of neurologic involvement in patients with HIV-1 infection. The presence of cICAM-1 in CSF of patients with constitutional disease (CDC group IV-A), however, suggests that the intrathecal release of this molecule may precede the onset of clinically manifested neurologic disease.

The correlation between cICAM-1 and levels of TNF-α presented here extends earlier *in vitro* observations, which reported an increased expression of ICAM-1 on brain microvascular endothelial cells after activation of the cell with TNF-α or other proinflammatory cytokines in a dose-dependent manner.[35] Further evidence was presented recently that the TNF-α mediated increase in vascular permeability and oedema are instituted by ICAM-1-dependent mechanisms[36]—a fact that may explain the significant correlation of cICAM-1 with both TNF-α levels and blood-brain barrier damage in our patients.

The importance of our results is also suggested by recent *in vivo* studies, which detected abundant expression of ICAM-1 on brain endothelial cells in inflammatory/infectious CNS diseases, such as herpes simplex encephalitis and in active multiple sclerosis plaques.[15] Interestingly, ICAM-1 is poorly expressed by CNS endothelial cells in animals without an intrathecal inflammatory process,[37] but ICAM-1 expression is upregulated during the initial phase of chronic relapsing allergic encephalomyelitis in association with inflammatory cell invasion.[29]

In conclusion, these results suggest that cICAM-1 may be important in the pathogenesis of inflammatory changes within the intrathecal compartment of HIV-1 infected patients. The correlation of cICAM-1 with both TNF-α concentrations and the degree of blood-brain barrier damage supports a functional role. It has to be noted, however, that the molecular events regulating cellular migration to the intrathecal compartment are multifactorial and involve several adhesion molecules in addition to ICAM-1. Analysing the dynamics of these molecules is crucial to evaluate the inflammatory cell invasion of CNS in HIV-1 infection.

### References

1. McArthur JC. Neurologic complications of AIDS. *Medicine* 1987; **66:** 407–437.
2. Levy RM, Breseden DE. Central nervous system dysfunction in acquired immunodeficiency syndrome. *J Acquir Immune Def Synd* 1988; **1:** 41–64.
3. Matsuyama T, Kobayashi N, Yamamoto N. Cytokines and HIV infection: is AIDS a tumor necrosis factor disease? *AIDS* 1991; **5:** 1405–1417.
4. Gray F, Lescs M-C, Keohane C, *et al.* Early brain changes in HIV infection: neuropathological study of 11 HIV seropositive, non-aids cases. *J Neuropathol Exp Neurol* 1992; **51:** 177–185.
5. Gherardi R, Mhiri C, Baudrimont M, Berry JP, Poirier, J. Iron pigment deposits, small vessel vasculitis and erythro-phagocytosis in the muscle of HIV-infected patients. *Human Pathol* (in press).
6. Nawroth PP, Stern DM. Modulation of endothelial cell hemostatic properties by tumor necrosis factor. *J Exp Med* 1986; **163:** 740–746.
7. Kahaleh MB, Smith EA, Soma Y, LeRoy EC. Tumor necrosis factor inhibits endothelial cell growth. *Clin Immunol Immunopathol* 1988; **49:** 261–272.
8. Sharief MK, Ciardi M, Thompson EJ, *et al.* Tumour necrosis factor-alpha mediates blood-brain barrier damage in HIV-1 infection of the central nervous system. *Mediators Inflamm* 1992; **1:** 191–196.
9. Osborn L. Leukocyte adhesion to endothelium in inflammation. *Cell* 1990; **62:** 3–6.
10. Singer SJ. Intercellular communication and cell–cell adhesion. *Science* 1992; **255:** 1671–1677.
11. Cotran RS, Gimbrone MA, Bevilacqua MP, Mendrick DL, Pober JS. Induction and detection of a human endothelial activation antigen *in vivo*. *J Exp Med* 1986; **164:** 661–667.
12. Makgoba MW, Sanders ME, Ginther GE, *et al.* ICAM-1 a ligand for LFA-1-dependent adhesion of B, T, and myeloid cells. *Nature* 1988; **331:** 86–88.
13. Diamond MS, Staunton DE, de Fougerolles AR, *et al.* ICAM-1 (CD54): a counter-receptor for Mac-1 (CD11a/CD18). *J Cell Biol* 1990; **3:** 3129–3141.
14. Argenbright LW, Barton RW. Interactions of leukocyte integrins with intercellular adhesion molecule 1 in the production of inflammatory vascular injury *in vivo*. The Shwartzman reaction revised. *J Clin Invest* 1992; **89:** 259–272.
15. Sobel R, Mitchell ME, Fondren G. Intercellular adhesion molecule-1 (ICAM-1) in cellular immune reactions in the human central nervous system. *Am J Pathol* 1990; **136:** 1309–1314.
16. Seth R, Raymond FD, Makagoba MW. Circulating ICAM-1 isoform: diagnostic prospects for inflammatory and immune disorders. *Lancet* 1991; **338:** 83–84.
17. Rothlein R, Mainolfi EA, Czajkowski M, Marlin SD. A form of circulating ICAM-1 in human serum. *J Immunol* 1991; **147:** 3788–3793.
18. Pober JS, Lapierre LA, Stolpen AH, *et al.* Activation of cultured human endothelial cells by recombinant lymphotoxin: comparison with tumor necrosis factor and interleukin species. *J Immunol* 1987; **138:** 3319–3325.
19. Koopman G, Pals ST. Cellular interactions in the germinal centre: role of adhesion receptors and significance for the pathogenesis of AIDS and malignant melanoma. *Immunology* 1992; **126:** 21–45.
20. Centers for disease control. Revision for the CDC surveillance case definition for acquired immunodeficiency syndrome. *Morbid Mortal Week Rep* 1987; **36:** 3s–15s.
21. Prieto J, Takei F, Gendelman R, Christenson B, Biberfeld P, Patarroyo M. MALA-2, mouse homologue of human adhesion molecule ICAM-1 (CD54). *Eur J Immunol* 1989; **19:** 1551–1557.
22. Neumann B, Ritter K, Felgenhauer K. Immunoblot for densitometric estimation of antibodies (IDEA): a useful method for quantification of intrathecally produced antibodies against individual antigens in infectious diseases of the central nervous system. *J Neuroimmunol* 1991; **34:** 173–179.
23. Mitchie HR, Manogue KR, Spriggs DR, *et al.* Detection of circulating tumor necrosis factor after endotoxin administration. *N Engl J Med* 1988; **318:** 1481–1486.
24. Tibbling G, Link H, Ohman S. Principles of albumin and IgG analyses in neurological diseases. I. Establishment of reference values. *Scand J Clin Lab Invest* 1977; **37:** 285–390.
25. Sharief MK, Ciardi M, Thompson EJ. Blood-brain barrier damage in patients with bacterial meningitis: association with tumor necrosis factor-alpha but not interleukin-1beta. *J Infect Dis* 1992; **166:** 350–358.
26. Cross AH, Cannella B, Brosnan CF, Raine CS. Homing to central nervous system vasculature by antigen-specific lymphocytes. I. Localization of 14C-labeled cells during acute, chronic, and relapsing experimental allergic encephalomyelitis. *Lab Invest* 1990; **63:** 162–170.
27. Raine CS, Cannella B, Duijvestijn AM, Cross AH. Homing to central nervous system vasculature by antigen-specific lymphocytes. II. Lymphocyte/endothelial cell adhesion during the initial stages of autoimmune demyelination. *Lab Invest* 1990; **63:** 476–489.
28. Wawryk SO, Novotny JR, Wicks IP, *et al.* The role of LFA-1/ICAM-1 interaction in human leukocyte homing and adhesion. *Immunol Rev* 1989; **108:** 135–161.
29. Cannella B, Cross AH, Raine CS. Upregulation and coexpression of adhesion molecules correlate with relapsing autoimmune demyelination in the central nervous system. *J Exp Med* 1990; **172:** 1521–1524.
30. Lassmann H, Rossler K, Zimprich F, Vass K. Expression of adhesion molecules and histocompatibility antigens at the blood-brain barrier. *Brain Pathol* 1991; **1:** 115–123.
31. Rossler K, Neuchrist C, Kitz K, Scheiner O, Kraft D, Lassmann H. Expression of leucocyte adhesion molecules at the human blood-brain barrier. *J Neurosci Res* 1992; **31:** 365–374.
32. Mizusawa H, Hirano A, Llena JF, Shintaku M. Cerebrovascular lesions in aquired immune deficiency syndrome. *Acta Neuropathol* 1988; **76:** 451–457.
33. Brew BJ, Bhalla RB, Fleisher M, *et al.* Cerebrospinal fluid beta2 microglobulin in patients infected with human immunodeficiency virus. *Neurology* 1989; **39:** 830–834.
34. Felgenhauer K, Luer W, Poser S. Chronic HIV encephalitis. *J Neuroimmunol* 1988; **20:** 141–144.
35. Fabry Z, Waldschmidt MM, Hendrickson D, *et al.* Adhesion molecules on murine brain microvascular endothelial cells: expression and regulation of ICAM-1 and Lgp 55. *J Neuroimmunol* 1992; **36:** 1–11.
36. Lo SK, Everitt J, Gu J, Malik AB. Tumor necrosis factor mediates experimental pulmonary edema by ICAM-1 and CD18-dependent mechanisms. *J Clin Invest* 1992; **89:** 981–988.
37. Wilcox RP, Ward AM, Evans A, Baker D, Rothlein R, Turk JL. Endothelial cell expression of the intercellular adhesion molecule-1 (ICAM-1) expression is upregulated in the central nervous system of guinea pigs during acute and chronic relapsing experimental allergic encephalopathy. *J Neuroimmunol* 1990; **30:** 43–51.

ACKNOWLEDGEMENTS. Dr M. Ciardi is supported by a grant from Istituto Superiore di Sanita, Italy. We thank June Smalley for her assistance in preparation of the manuscript.

**Received 23 June 1992; accepted in revised form 28 July 1992**

## References

[PDF_00553] Argenbright L. W., Barton R. W. (1992). Interactions of leukocyte integrins with intercellular adhesion molecule 1 in the production of inflammatory vascular injury in vivo. The Shwartzman reaction revisited.. J Clin Invest.

[PDF_00610] Brew B. J., Bhalla R. B., Fleisher M., Paul M., Khan A., Schwartz M. K., Price R. W. (1989). Cerebrospinal fluid beta 2 microglobulin in patients infected with human immunodeficiency virus.. Neurology.

[PDF_00601] Cannella B., Cross A. H., Raine C. S. (1990). Upregulation and coexpression of adhesion molecules correlate with relapsing autoimmune demyelination in the central nervous system.. J Exp Med.

[PDF_00545] Cotran R. S., Gimbrone M. A., Bevilacqua M. P., Mendrick D. L., Pober J. S. (1986). Induction and detection of a human endothelial activation antigen in vivo.. J Exp Med.

[PDF_00590] Cross A. H., Cannella B., Brosnan C. F., Raine C. S. (1990). Homing to central nervous system vasculature by antigen-specific lymphocytes. I. Localization of 14C-labeled cells during acute, chronic, and relapsing experimental allergic encephalomyelitis.. Lab Invest.

[PDF_00551] Diamond M. S., Staunton D. E., de Fougerolles A. R., Stacker S. A., Garcia-Aguilar J., Hibbs M. L., Springer T. A. (1990). ICAM-1 (CD54): a counter-receptor for Mac-1 (CD11b/CD18).. J Cell Biol.

[PDF_00615] Felgenhauer K., Lüer W., Poser S. (1988). Chronic HIV encephalitis.. J Neuroimmunol.

[PDF_00568] Koopman G., Pals S. T. (1992). Cellular interactions in the germinal center: role of adhesion receptors and significance for the pathogenesis of AIDS and malignant lymphoma.. Immunol Rev.

[PDF_00524] Levy R. M., Bredesen D. E. (1988). Central nervous system dysfunction in acquired immunodeficiency syndrome.. J Acquir Immune Defic Syndr.

[PDF_00620] Lo S. K., Everitt J., Gu J., Malik A. B. (1992). Tumor necrosis factor mediates experimental pulmonary edema by ICAM-1 and CD18-dependent mechanisms.. J Clin Invest.

[PDF_00548] Makgoba M. W., Sanders M. E., Ginther Luce G. E., Dustin M. L., Springer T. A., Clark E. A., Mannoni P., Shaw S. (1988). ICAM-1 a ligand for LFA-1-dependent adhesion of B, T and myeloid cells.. Nature.

[PDF_00526] Matsuyama T., Kobayashi N., Yamamoto N. (1991). Cytokines and HIV infection: is AIDS a tumor necrosis factor disease?. AIDS.

[PDF_00522] McArthur J. C. (1987). Neurologic manifestations of AIDS.. Medicine (Baltimore).

[PDF_00581] Michie H. R., Manogue K. R., Spriggs D. R., Revhaug A., O'Dwyer S., Dinarello C. A., Cerami A., Wolff S. M., Wilmore D. W. (1988). Detection of circulating tumor necrosis factor after endotoxin administration.. N Engl J Med.

[PDF_00531] Nawroth P. P., Stern D. M. (1986). Modulation of endothelial cell hemostatic properties by tumor necrosis factor.. J Exp Med.

[PDF_00541] Osborn L. (1990). Leukocyte adhesion to endothelium in inflammation.. Cell.

[PDF_00563] Pober J. S., Lapierre L. A., Stolpen A. H., Brock T. A., Springer T. A., Fiers W., Bevilacqua M. P., Mendrick D. L., Gimbrone M. A. (1987). Activation of cultured human endothelial cells by recombinant lymphotoxin: comparison with tumor necrosis factor and interleukin 1 species.. J Immunol.

[PDF_00594] Raine C. S., Cannella B., Duijvestijn A. M., Cross A. H. (1990). Homing to central nervous system vasculature by antigen-specific lymphocytes. II. Lymphocyte/endothelial cell adhesion during the initial stages of autoimmune demyelination.. Lab Invest.

[PDF_00557] Seth R., Raymond F. D., Makgoba M. W. (1991). Circulating ICAM-1 isoforms: diagnostic prospects for inflammatory and immune disorders.. Lancet.

[PDF_00587] Sharief M. K., Ciardi M., Thompson E. J. (1992). Blood-brain barrier damage in patients with bacterial meningitis: association with tumor necrosis factor-alpha but not interleukin-1 beta.. J Infect Dis.

[PDF_00536] Sharief M. K., Ciardi M., Thompson E. J., Sorice F., Rossi F., Vullo V., Cirelli A. (1992). Tumour necrosis factor-alpha mediates blood-brain barrier damage in HIV-1 infection of the central nervous system.. Mediators Inflamm.

[PDF_00543] Singer S. J. (1992). Intercellular communication and cell-cell adhesion.. Science.

[PDF_00584] Tibbling G., Link H., Ohman S. (1977). Principles of albumin and IgG analyses in neurological disorders. I. Establishment of reference values.. Scand J Clin Lab Invest.

[PDF_00598] Wawryk S. O., Novotny J. R., Wicks I. P., Wilkinson D., Maher D., Salvaris E., Welch K., Fecondo J., Boyd A. W. (1989). The role of the LFA-1/ICAM-1 interaction in human leukocyte homing and adhesion.. Immunol Rev.

[PDF_00623] Wilcox C. E., Ward A. M., Evans A., Baker D., Rothlein R., Turk J. L. (1990). Endothelial cell expression of the intercellular adhesion molecule-1 (ICAM-1) in the central nervous system of guinea pigs during acute and chronic relapsing experimental allergic encephalomyelitis.. J Neuroimmunol.

